# The Dose of Nitroglycerine

**Published:** 1892-10

**Authors:** 


### THE DOSE OF NITROGLYCERINE.

At a recent meeting of the Practitioners' Society in New York, Dr. George F. Peabody stated that the negative results often obtained in the use of nitroglycerine for high arterial tension could be changed by increasing the dose. Unless some contraindication were present, he would push the dose of the drug to the point of producing some specific effect. The ordinary dose is put down as 1-100 of a grain, but he has often had to exceed this before any effect could be noticed. In one of his cases, patient sixty years of age, with diffuse nephritis, mitral insufficiency and general arterial sclerosis, extending over a period of four or five years, he began with the usual dose and gradually increased until his patient took two grains every two hours, or twenty-four grains per day. It was only under this latter dosage that the dyspnœa disappeared and strength increased sufficiently to enable the patient to go about. In another case, with high-tension pulse, hypertrophy of left ventricle, with occasional attacks of profuse and painful vomiting, he began with 1-100 of a grain and increased up to one grain every three hours, day and night. During the attacks of vomiting the drug was given in the same doses hypodermically. Condition of the pulse was much improved. Vomiting had to be controlled by morphine.

Dr. Kinnicutt reported a case of general arterial sclerosis with high-tension pulse and distressing spmptoms, in which the dose of nitroglycerine had to be carried up to three grains and a half every hour during the day. These large doses improved and relieved all the bad symptoms. The drug, he thought, ought not to be abandoned because in ordinary doses it failed to produce the desired effects. The dose should be increased until desired physiological effects were obtained.

